# Correction: Motor variability during resistance training: Acceleration signal as intensity indicator

**DOI:** 10.1371/journal.pone.0320873

**Published:** 2025-03-25

**Authors:** Miguel López-Fernández, Fernando García-Aguilar, Pablo Asencio, Carla Caballero, Francisco J. Moreno, Rafael Sabido

The images for Figs 1 and 2 are incorrectly switched. The image that appears as Fig 1 should be Fig 2, and the image that appears as Fig 2 should be Fig 1. The figure captions appear in the correct order. The authors have provided a corrected version of figures here.

**Fig 1:**
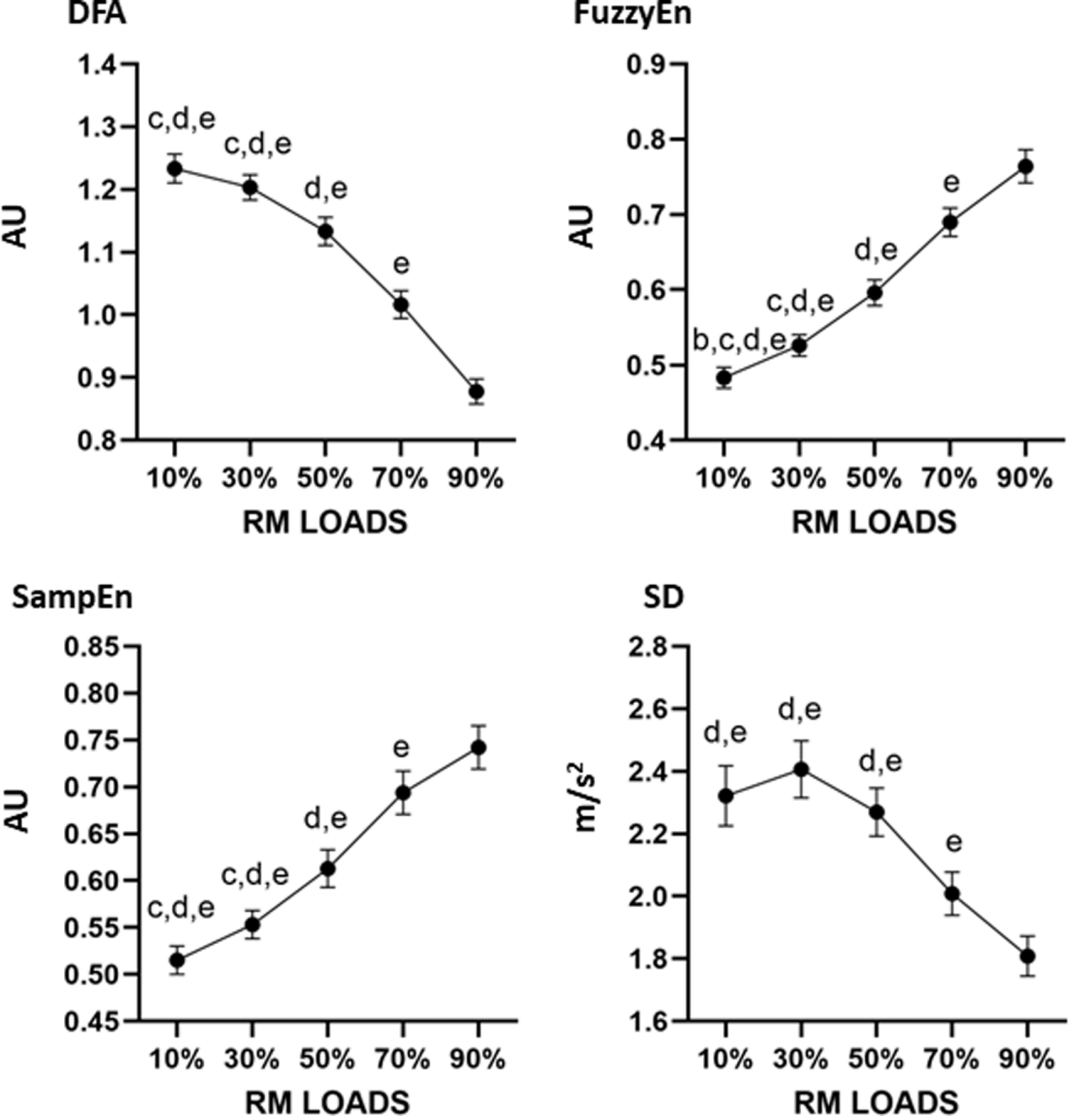
Mean differences between the different percentages of loads for the measurements with the IMU device. AU: arbitrary units. Letter corresponds to the different load comparisons: b = differences versus 30%; c = difference versus 50%; d = difference versus 70%; e = difference versus 90%.

**Fig 2:**
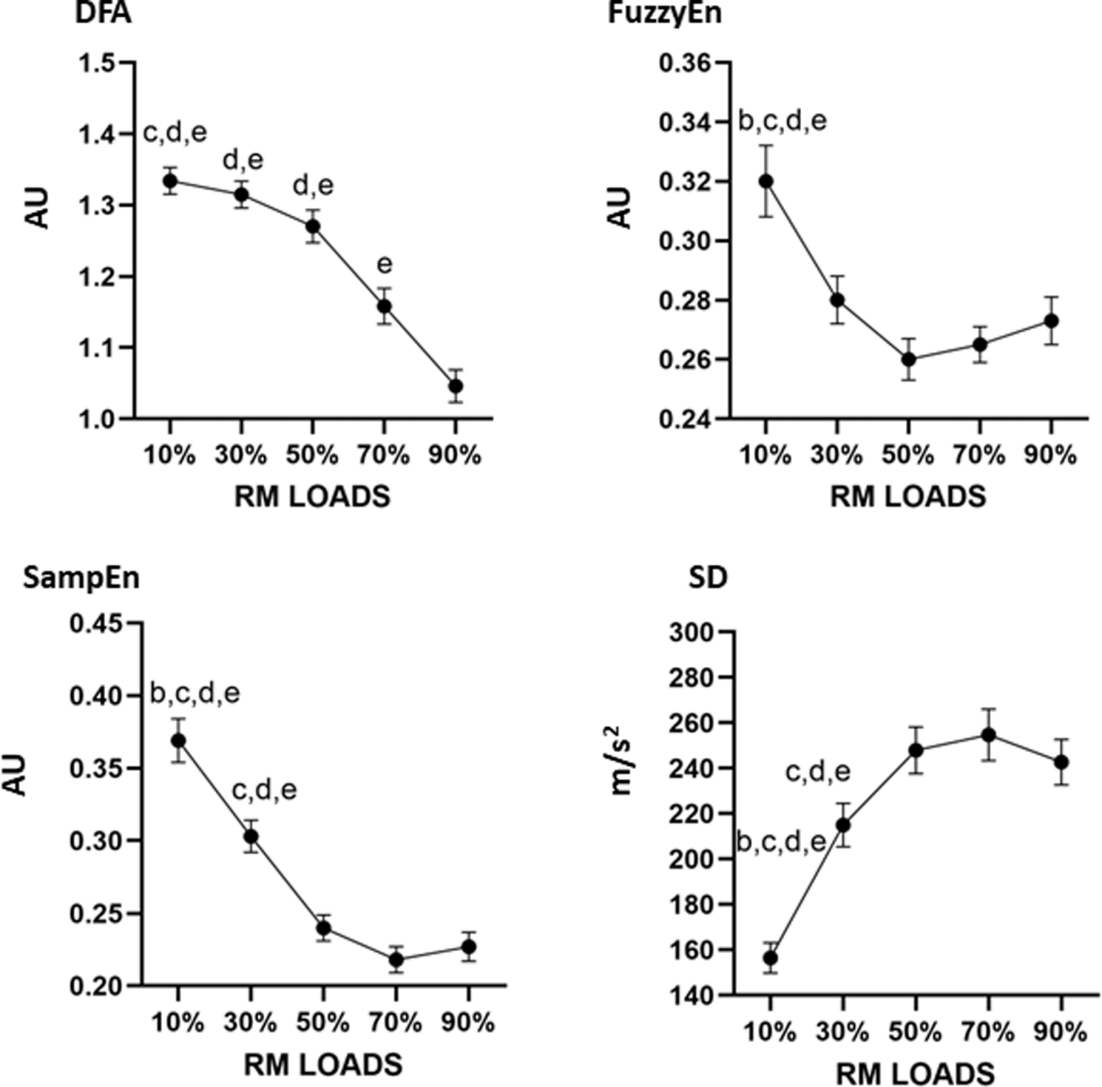
Mean differences between the different percentages of loads for the measurements with the force platform. AU: arbitrary units. Letter corresponds to the different load comparisons: b = differences versus 30%; c = versus 50%; d = versus 70%; e = difference versus 90%.
