# Supplementary material for: Motor variability during resistance training: Acceleration signal as intensity indicator
Source: PLoS One. 2024 Sep 19;19(9):e0307949. doi: 10.1371/journal.pone.0307949 (PMC11412665; doi:10.1371/journal.pone.0307949)
Supplement: Attachment — Submitted filename: Response to Reviewers.docx [file pone.0307949.s001.docx]

Dear Academic Editor, Ms. Danica Janicijevic,

You can find enclosed the revision file “PONE-D-24-09826” to the following manuscript that has been submitted by Fernando García-Aguilar and co-authors for consideration for publication in the *PLOS ONE*, entitled **"Motor variability during resistance training: acceleration signal as intensity indicator".**

We appreciate the reviewer’s thoughtful comments, and we hope he/she will agree that the updated version is much stronger as a result. In all areas covered by the reviewer, we have tried to deal with his/her concerns as fully as possible. Below we present a point-by-point overview of the changes that we have made to the manuscript as requested by the reviewers. Those changes are highlighted in red colour in the manuscript.

Thank you for your consideration, and we look forward to hearing from you soon.

03/07/2024

Sincerely,

Fernando García Aguilar

PhD Student at Miguel Hernández University.

Edificio CID, Av. de la Universidad s/n, 03202 Elche, Alicante.

E-mail address Fernando.garciaa@umh.es

**Authors’ response (AR):** We would like to thank the reviewers for their helpful advice and suggestions regarding this manuscript. We found their criticisms and recommendations very constructive. We appreciate the work done in revising this manuscript, and we have responded to your concerns point by point. In the new version of the manuscript, changes have been marked in red.

**Reviewer 1**

The research presented by the authors is very interesting and important for the proper motor preparation of players. The authors fulfilled the criteria for scientific works. In the reviewer's opinion, greater care should be taken in the selection of current literature in the future. The authors rightly pointed out the limitations of the research conducted, which will translate into better performance in subsequent studies.

**AR:** Thank you very much for your kind words and for your appreciation of our work. We are pleased to know that you find our study relevant and important for the scientific field and its applicability. We appreciate your support and are grateful for your positive comments.

**Reviewer 2**

*1. It is understood that these four methods are important analyses for accurately analyzing variability in load and intensity during strength training. However, for their application to be more understandable and usable, it is necessary to highlight the loads where variability is detected and not detected in the conclusion section. Additionally, the measures to be taken during the application (for example, the lack of difference between 10% and 30% in DFA) should be addressed. In short, how can coaches or athletes shape their strength training practices after reading this paper? This topic needs to be emphasized*.

**AR:** Thank you for this suggestion. We have added your suggestion in the conclusions section in line 277 indicating “The records of IMUs using non-linear measurements (DFA, FuzzyEn, SampEn) indicate an increase in motion complexity with an elevated percentage load (above 30%). However, at low loads (below 30%) in the IMU, no differences are found (although there is the same tendency to increase complexity), as it is more difficult to find differences when the intensity of the task is less challenging for the system. In the records obtained with a force platform, the complexity is observed to decrease, with changes between loads detected by DFA analysis”.

*2. How can coaches or athletes shape their strength training practices after reading this paper? This topic needs to be emphasized. In this sense, what should coaches and athletes do with the data provided by these four methods regarding strength variability? Should they train at certain 1RM percentages with more units, or is it unnecessary to spend too much time at certain 1RM percentages? For quick adaptation, what should be considered in light of these four methods?*

**AR:** Thank you for this suggestion. In line 283, we have deleted "It appears" and added "These results indicate". In the same paragraph, but in line 284, we have added the following: “indicating that as the intensity of the load increases, the system is more compromised and increases its complexity to overcome the task. Therefore, the use of non-linear measures that analyze the complexity of movement through acceleration can be useful for coaches to know the internal load that the different load intensities used on their athletes place on the system. In this way, they will also be able to know the state in which the athlete measuring complexity with a same load in different days or sessions” to answer your suggestions on the applications of these results for coaches and practical applications. We have also changed the sentence from line 291 onwards and have added “while FuzzyEn and SampEn are sensitive through IMUs, so all these tools are useful for training practice”.

**Journal requirements**

*Section 2. Financial Disclosure: Please state what role the funders took in the study.*

**AR:** Thank you for the request on this point, we would like to say that “the funders had no role in study design, data collection and analysis, decision to publish, or preparation of the manuscript”

*Section 3. Acknowledgment: Please remove any funding-related text from the manuscript and let us know how you would like to update your Funding Statement.*

**AR:** We would like to express our gratitude for bringing this section of the text to our attention. In light of this, we have removed information on funding from the acknowledgements section and would like to update the Funding Statement to state that “This manuscript is related to a national project funded by the Ministerio de Ciencia e Innovación [PID2019-109632RB-I00]. The contribution of Miguel López-Fernández was funded by the Generalitat Valenciana, Spain [grant number: CIACIF/2021/452]. The contribution of Fernando García-Aguilar was supported by the Generalitat Valencia, Spain [grant number: ACIF/2021/159]. The contribution of Pablo Asencio was supported by the Ministerio de Ciencia e Innovación [PRE2020-091858]. These grants have made it possible to carry out this research.

The funders had no role in study design, data collection and analysis, decision to publish, or preparation of the manuscript”.

*Section 5. Ethics statement: Your ethics statement should only appear in the Methods section of your manuscript. If your ethics statement is written in any section besides the Methods, please delete it from any other section.*

**AR:** Thank you for the indication on this point. The section on the ethics statement has been removed, and we have written in the methods section the following “Prior to participation, each subject provided written informed consent, which was approved by the ethics committee of the University (PID2019-109632RB-100), and which adhered to the Declaration of Helsinki. The Office of Responsible Research (OIR), as the University's ethics committee, approves the research ethics statement under register 2019.417.E.OIR; 2020.34.E.OIR and reference DCD.RSS.02.19”.
